# Using deep learning models to decode emotional states in horses

**DOI:** 10.1038/s41598-025-95853-7

**Published:** 2025-04-23

**Authors:** Romane Phelipon, Lea Lansade, Misbah Razzaq

**Affiliations:** INRAE, CNRS, Université de Tours, PRC, 37380 Nouzilly, France

**Keywords:** Animal behaviour, Machine learning

## Abstract

In this study, we explore machine learning models for predicting emotional states in ridden horses. We manually label the images to train the models in a supervised manner. We perform data exploration and use different cropping methods, mainly based on Yolo and Faster R-CNN, to create two new datasets: 1) the cropped body, and 2) the cropped head dataset. We train various convolutional neural network (CNN) models on both cropped and uncropped datasets and compare their performance in emotion prediction of ridden horses. Despite the cropped head dataset lacking important regions like the tail (commonly annotated by experts), it yields the best results with an accuracy of 87%, precision of 79%, and recall of 97%. Furthermore, we update our models using various techniques, such as transfer learning and fine-tuning, to further improve their performance. Finally, we employ three interpretation methods to analyze the internal workings of our models, finding that LIME effectively identifies features similar to those used by experts for annotation.

## Introduction

Animal welfare is becoming an increasingly important social concern, especially with respect to animals utilized by humans. Equestrian sports are no exception, and numerous horse riding practices are currently criticized for being detrimental to horse comfort^1^. However, no tool has been proposed to evaluate horses while they are being ridden. In order to identify the horse’s state of well-being when ridden, certain indicators based on behaviors, posture, and facial expressions must be taken into account^2,3^. For example, tail swishing behavior indicates a state of discomfort in the horse when ridden and is often synchronized with the use of spurs. In addition, the head-behind-the-vertical posture is recognized as a sign of the horse’s discomfort^2^. It is a well-established fact that in addition to behavior and posture, facial expressions can reflect emotional states in horses^4^. Therefore, certain facial cues, such as the opening of the eyes or mouth and the position of the ears, provide information about the comfort level of the ridden horse^3^. However, measuring these indicators is challenging and requires sustained observation by individuals with experience in the field. In fact, it is generally necessary to carefully watch videos of horses and manually code each behavior, posture, or facial expression. This process is highly time-consuming, as it is done manually and sometimes even frame by frame (e.g., in the case of facial expressions^4–9^). In addition, it involves a subjective element, often necessitating that two individuals independently review the same video to verify the consistency of the measurements. While manual analysis remains necessary for labeling and validation, its limitations highlight the need for complementary automated methods.

Artificial intelligence (AI)-based methods have gained prominence in recent years, successfully applied in areas such as image recognition, robotics, speech recognition, and life sciences. In this context, an automated deep learning system that detects regions of interest, such as facial expressions and posture, would significantly aid in promoting animal welfare. Although preliminary studies and proofs of concept have been conducted on the potential of artificial intelligence to detect lameness^10^, facial and head movements^11^, pain^12,13^, and, more generally, the horse’s emotional state^14^, there is still a need for a demonstration of artificial intelligence capable of assessing the well-being of the ridden horse. Studying ridden horses appears essential, as this could assist in the promotion of ethical conduct, the prevention of accidents through enhanced safety measures, and, more generally, the improvement of equine welfare. In one study^13^, the authors built classification models using convolutional neural networks (CNNs) based on facial features. These features were annotated using the horse grimace scale method. This study was based on the assessment of the facial expressions of seven horses undergoing castration, which were filmed two days before and four days after the procedure. They selected 3, 000 images by visual inspection out of 185,672 extracted frames from videos. Finally, this dataset was divided into three subsets based on different features: 1) ears, 2) eyes, and 3) chewing muscles, mouths, and nostrils. They obtained an accuracy of 75.8% for classifying pain into three categories: not present, moderately present, and obviously present. However, for the binary classification, i.e., the presence or absence of pain, they achieved an accuracy of 88.3%. In another study^14^, the authors developed a detector to recognize horses in images and a classifier to predict the emotion of the detected horse. To train and test the system, a dataset of 440 images was collected from private sources, with each image labeled with one of four emotional markers: alarmed, annoyed, curious, or relaxed. There were 110 images per emotion, and the dataset was divided into 400 training images and 40 test images. Their model achieved an accuracy of 65% on the testing set.

As compared to the aforementioned methods, in this work, we used a combination of labeling methods: HGS^15^ and RHpE^16^, instead of using a single method. This approach enables us to obtain information not only on facial emotions but also from other body parts of horses. Previous results have shown the need to crop the images in the dataset to allow the model to focus on the crucial elements of the image while reducing the influence of the background on prediction accuracy. We used pre-trained YOLO and Faster R-CNN to identify and crop the horse’s body and the horse’s head. We used several pre-processing methods to improve the quality of the data, such as data augmentation or resolution augmentation. Then, for the classification step, we developed a model from scratch, referred to as the starting model. We also used transfer learning and fine-tuning techniques using different backbone architectures, such as VGG16 and ResNet50. For hyperparameter optimization, we used a Bayesian search algorithm. Finally, we concatenated the best performing models (VGG16 and Xception) and obtained better results than individual models, a recall of 97% and an accuracy of 87% in the test set. Given the well-known black-box nature of deep learning models, we employed several interpretation techniques to identify the key features used by our models. We generated explanations for different predictions and compared the outputs of various interpretation methods. By analyzing these explanations, we further underscored the importance of developing robust interpretation techniques.

## Methods

### Dataset

Our dataset consisted of 1, 036 images of horses divided into two classes: comfortable (546) and uncomfortable (490). The images used in this study were obtained in an opportunistic manner; consequently, it is important to emphasize that no horse was specifically used or constrained for the purposes of this study. The images were collected from multiple sources, including public sources such as online image databases such as Pixabay, Pexels, and Freepik. Additionally, private sources were utilized, including photographs captured directly during equestrian events and images provided by private photo libraries of equestrian organizations. The dataset included images of more than 250 different horses and ponies of various breeds (French saddle, Totteur, Quarter Horse, Thoroughbred, Shetland and others), always ridden and in various disciplines (leisure, trail riding, horse racing, show jumping, dressage, eventing, western riding, and others).

We divided the dataset into three distinct subsets: the training set (70%), the validation set (15%), and the test set (15%). The training set was used to train the machine learning model, optimizing model parameters to minimize loss. The validation set was used to fine-tune the model, typically by adjusting hyperparameters. The test set was a part of the dataset that the model had never encountered during training or validation and was used as an unbiased assessment of the model’s performance.

### Annotations

A doctoral student in ethology specializing in the identification of horse emotions and a technician in ethology selected the images. The images came either from private sources or from the internet on copyright-free image sites. Images in the comfortable and uncomfortable category were selected according to features described in previous studies^2,3^ and depending on the state of five key points listed in Table 1. Ears forward, erect and parallel with pinnae facing forward^3^ was considered a comfortable feature as opposed to both ears backward, which is commonly considered to be a negative state of the ridden horse^2,17^. Open, round, and tension-free eyes were considered a comfortable feature, whereas “almond-shaped eyes with tension of musculus levator anguli oculi medialis”^3^ (i.e., tension above the eyes) were considered an uncomfortable feature as suggested in a study on the development of ridden horses ethogram focused on the facial expressions^3^. The opening of the horses’ mouths was also evaluated. If the mouth was closed, it was considered comfortable. If the mouth was open, it was considered uncomfortable, as studies have shown that horses with more constraints when being ridden or those with musculoskeletal pain open their mouths more often^17,18^. Head behind the vertical is known as a practice with negative effects on horses^1,19^. Thus, a head behind the vertical was considered an uncomfortable feature; if not, it was considered a comfortable feature. Tail swishing is a behavior that is generally expressed by horses when they feel uncomfortable while being ridden or as a conflict behavior^2^, so the feature was therefore considered as part of the uncomfortable category. Basal tail with no swishing was considered as part of the comfortable category. If horses expressed in images at least 2 features belonging to the category uncomfortable, they were placed in this category. Horses in the category comfortable expressed the 5 comfortable features.

**Table 1:** Key points for annotating different emotional states in horses.

Comfortable	Uncomfortable
Forward ears	Backward ears
Open eyes without tension	Tension above the eyes Closed eyes or Half closed eyes Sclera exposed
Basal tail	Tail swishing
Closed mouth	Open mouth
Head not behind the vertical	Head behind the vertical

### Preprocessing

#### Image resolution

Our dataset contained both low-resolution and high-resolution images. Image resolution plays an important role in the accuracy of pattern recognition in neural networks. We verified the impact of higher resolutions on computational time and accuracy of the model. A smaller image resolution led to a reduction in training time, while increasing the resolution allowed the model to focus on smaller features (e.g., mouth opening). It was shown that increasing the size of the input image could improve the accuracy of the predictions up to a certain point^20^. Thus, a higher resolution did not always lead to better predictions. That is why we decided to fix the resolution to $256 \times 256$, which is generally employed in CNN-based methods.

#### Data augmentation

One of the most common techniques used in machine learning and computer vision for increasing the size and diversity of a training dataset is data augmentation. This technique applies different transformation methods to the images to improve the capacity of the model to generalize, i.e., prevent overfitting, and improve its performance on unseen data^21^. There are two main ways to perform data augmentation. One way is to increase the images (by performing certain transformations) within the dataset, resulting in an increase in the dataset itself. This method had a number of disadvantages, in particular because we needed to determine the number of images that could be generated from a single image to avoid generating images that were too close semantically. We also needed to separate our dataset into subsets beforehand to avoid data leakage by finding the same or similar images in several subsets, which distorted the results. An alternative is to use a second class of methods that apply a random combination of the transformations to the images during the training process. In our study, we employed the second method using the TensorFlow image augmentation function^22^.

#### Cropped dataset

Previous results indicated the need to crop the images in the dataset to enable the model to focus on the critical elements of the image while minimizing the influence of the background on prediction accuracy. In this study, we divided the cropped dataset into two subsets: 1) a horse body dataset and 2) a horse head dataset. To perform the cropping operation, we utilized YOLO and Faster R-CNN.

To create a horse body cropping dataset, we developed a pipeline using YOLOv8x^23^ to identify and crop only the horse bodies recognized by the model. However, the dataset included some images containing multiple horses. To avoid contamination of the final dataset, we applied a selection criterion based on the assumption that each image corresponds to a single horse. In cases where multiple horses were detected, we retained only the image of the horse’s body with the highest resolution. After a manual review, no incorrect cropping were identified, and only two images were excluded from the pipeline due to format issues.

To crop the horse’s head, we used a pretrained Faster R-CNN model^14^ capable of extracting the horse’s head from its body. A subsequent manual review identified errors in six images, including incorrect cropping or format issues.

### Models

#### Object detection algorithms

Faster Region-Based Convolutional Neural Networks (Faster R-CNN) is a two-stage object detection algorithm^24^. It uses a convolutional neural network (CNN) and a region proposal network (RPN) to identify and locate objects. It operates in two stages: 1) Feature Extraction: A CNN processes the input image to extract high-level features, and 2) Region Proposal and Classification: The RPN, a fully convolutional network, predicts object proposals and scores for each object’s position. It generates region proposals by sliding anchor windows over the feature map. For each anchor, the RPN predicts (1) the probability that the anchor contains an object and (2) adjustments to refine the anchor’s alignment with the ground-truth bounding box. These region proposals are then passed to the classifier, which assigns class labels and further refines the bounding box coordinates.

YOLO (You Only Look Once) is an extremely fast real-time one-stage object detector where a single neural network predicts multiple bounding boxes and their classification probabilities simultaneously^25^. YOLO divides the entire image into an $S \times S$ grid, and if the center of an object falls within a particular grid cell, that cell is responsible for detecting the object. Each grid cell predicts several bounding boxes along with their confidence scores and class labels. The confidence score indicates the likelihood that an object has been detected, and YOLO multiplies class probabilities by the confidence scores of the bounding boxes to produce final detections.

Faster R-CNN significantly outperformed earlier methods, such as the Viola-Jones algorithm, in terms of detection accuracy and frames per second (FPS)^26^. However, YOLO has demonstrated competitive or superior performance in specific scenarios, particularly those requiring real-time detection^27,28^. While YOLO is faster than two-stage detectors, it may struggle with smaller objects and overlapping detections.

#### CNN architecture

CNNs are a specialized type of feed-forward neural network inspired by human vision, primarily used for image classification, object detection, and clustering of similar images. They are structured around three main layer types^29^: convolutional layers for feature extraction, pooling layers for dimensionality reduction, and fully connected layers for classification. A convolutional layer performs element-wise multiplications using learnable filters or kernels (matrices of numbers) applied to the input data, summing the results to generate feature maps. Pooling layers, such as max-pooling or average-pooling, are then used to sample the feature maps, retaining only essential information and eliminating noise and redundancy. This process enables CNNs to be invariant to small spatial translations, as these have minimal impact on the output of the pooling operation^30^. Finally, fully connected layer(s) are employed after pooling to perform tasks such as classification or regression^31^.

**Fig. 1:**
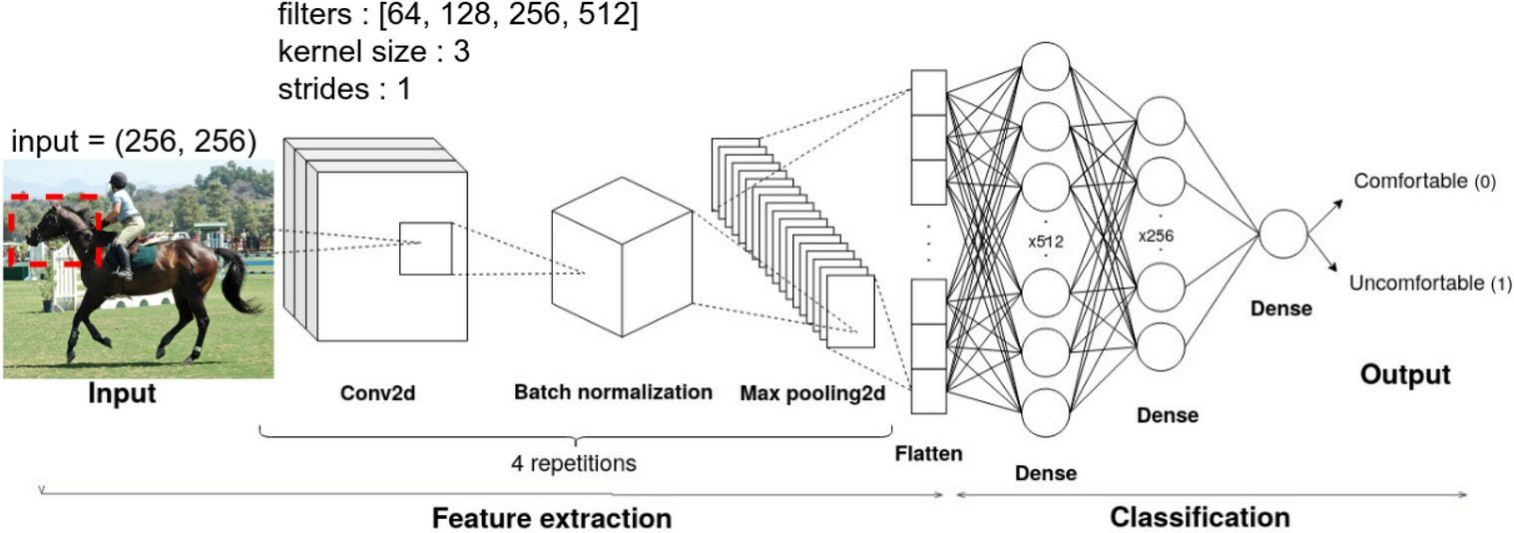
Starting CNN architecture representation.

We designed the CNN architecture by considering factors such as data representation, network topology, activation functions, and hyperparameter tuning (see Figure 1). For the feature extraction component, we developed four blocks, each consisting of convolutional layers, batch normalization layers, and max-pooling layers. For the classification component, we created three blocks comprising fully connected (dense) layers combined with dropout. A flatten layer was introduced to connect feature extraction to classification by reducing the features to a single-dimensional vector. To mitigate overfitting, we implemented dropout along with L1 or L2 regularization.

#### Hyperparameter tuning

Hyperparameter tuning involves finding the optimal set of hyperparameters for a machine learning model to maximize its performance according to a specified metric. Automated hyperparameter tuning methods use algorithms to search for these optimal values. In this study, we employed Bayesian optimization using the Hyperopt Python library^32,33^. The hyperparameters we adjusted include batch size, which was tested with values of 8, 16, and 32; filters, with values of 32, 64, 128, 256, and 512; kernel size, with values of 3 and 5; dropout rate, with values of 0.1, 0.2, 0.3, 0.4, and 0.5; and learning rate, with values of 0.01 and 0.0001.

#### Transfer learning and fine tuning

Transfer learning is a machine learning technique that allows knowledge acquired from training a model on one classification task, such as categorizing a specific class, to be applied to a different classification task involving another class. Using a transfer learning approach, we tested and compared several widely used pre-trained models: ResNet50, ResNet152, VGG16, VGG19, InceptionV3, Xception, and EfficientNetV2L. In our implementation, we replaced the original classification layers of these models with a custom classification head. Depending on the hyperparameter tuning, this head consisted of a flattening layer or a global average pooling layer, followed by a fully connected layer and dropout. We froze the weights of the feature extraction layers, retaining the pre-trained weights to accelerate learning and improve generalization.

To further enhance prediction performance, fine-tuning was employed. This process involved unfreezing specific feature extraction layers to adapt and specialize the model for recognizing particular horse features. Training only the classification layers does not generate feature maps directly influenced by the features of interest. Since the final convolutional layers contain high-level semantic information, they were unfrozen and retrained at the end of each training session (a learning phase that concludes when the model stops improving or begins to overfit). Conversely, the initial low-level layers, which excel at identifying fundamental features, were kept frozen to preserve their well-established weights.

#### Model stacking

A stacking model, also referred to as a stacked ensemble, is a machine learning technique that combines multiple base models to enhance overall predictive performance ^34^. In our approach, we integrated the best-performing models on our dataset, VGG16 and Xception, to create a stacked ensemble. First, these models were trained independently, and their outputs were combined as input to a meta-classifier. Once the stacked model was trained, it was used to make predictions on new, unseen data.

### Performance evaluation

Various metrics can be used to evaluate a model’s ability to generalize and its effectiveness in making predictions. In this study, we employed accuracy, precision, and recall to assess model performance. Accuracy measures the overall performance of the model by calculating the ratio of correct predictions (true positives and true negatives) to the total number of predictions (see equation 1).

1 $$\begin{aligned} Acc = \frac{TP \ + \ TN}{TP \ + \ TN \ + \ FP \ + \ FN} \end{aligned}$$

Here, TP (true positive) represents the number of instances correctly predicted as positive by the model (e.g., the emotional state is comfortable, and the model’s prediction is also comfortable). TN (true negative) denotes the number of instances correctly predicted as negative by the model (e.g., the emotional state is uncomfortable, and the model’s prediction is also uncomfortable). FP (false positive) is the number of instances incorrectly predicted as positive by the model (e.g., the emotional state is uncomfortable, but the model predicts comfortable). FN (false negative) is the number of instances incorrectly predicted as negative by the model (e.g., the emotional state is comfortable, but the model predicts uncomfortable).

Precision is calculated as the ratio of correctly classified positive samples to the total number of samples classified as positive. In this study, it represents the percentage of correct comfortable predictions among all predictions labeled as comfortable (see equation 2).

2 $$\begin{aligned} Precision = \frac{TP}{TP \ + \ FP} \end{aligned}$$

Recall was calculated as the proportion of actual positive samples that were identified correctly. In this study, it represents the percentage of correct predictions of comfortable among all actual comfortable emotional states (see equation 3).

3 $$\begin{aligned} Recall = \frac{TP}{TP \ + \ FN} \end{aligned}$$

### Interpretation methods

Machine learning models have made significant advancements in recent years, leading to the development of sophisticated models capable of tackling complex tasks with exceptional accuracy. However, as these models grow more complex and opaque, their interpretability becomes a critical concern. In this study, we employed state-of-the-art methods such as LIME (Local Interpretable Model-Agnostic Explanations)^35^, SHAP (SHapley Additive exPlanations)^36^, and Grad-CAM (Gradient-weighted Class Activation Mapping) ^37^ to gain insights into the inner workings of the proposed models. These methods help explain how the models make predictions by identifying key features or regions of interest.

## Results

### Preprocessing

**Fig. 2:**
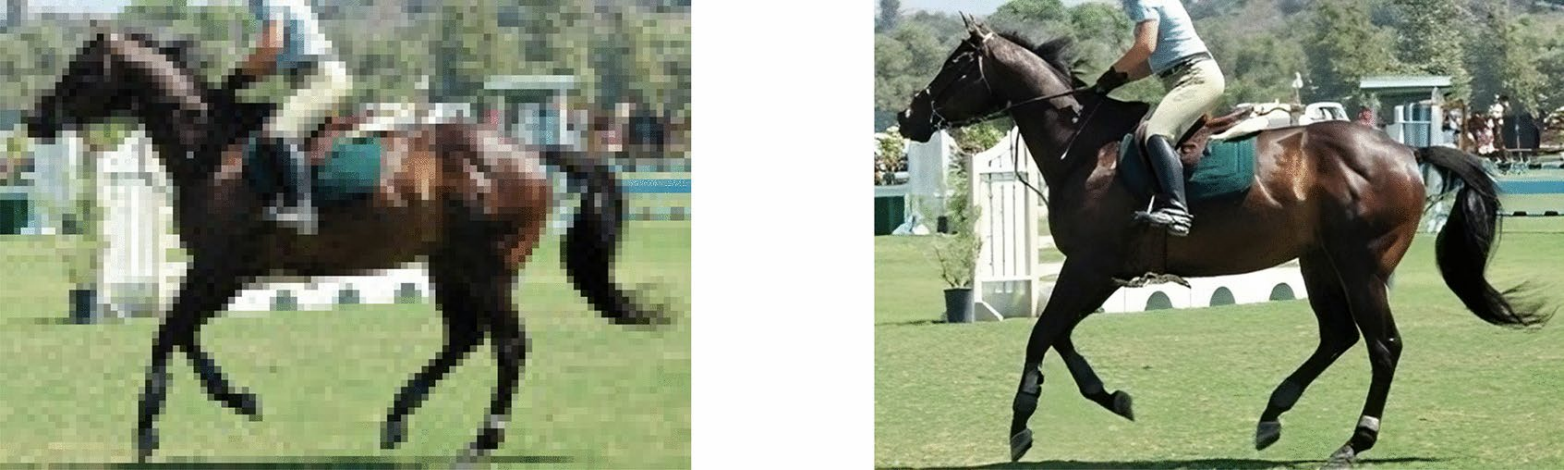
A horse images from our dataset going through the resolution augmentation.

Our dataset contained images with both high and low resolutions. Rescaling low-resolution images may have resulted in information loss. To address this issue, we tested various resolution augmentation techniques on our dataset. A manual review was conducted to select the method that provided the best resolution improvement without distorting the horse’s features. An example of the resolution augmentation applied to one image is shown in Figure 2.

**Fig. 3:**
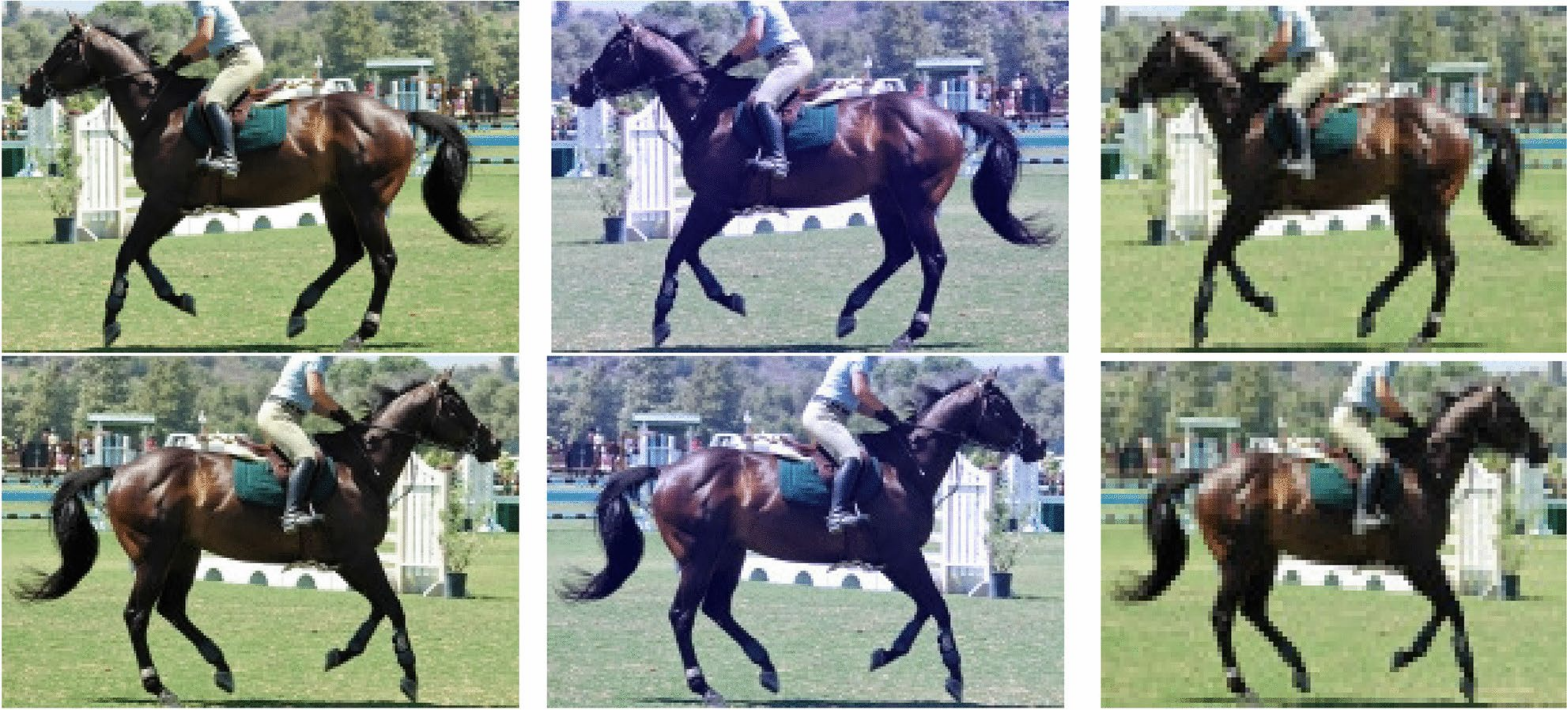
Examples of some data augmentation.

We applied various image transformation techniques, including rotation, scaling, flipping, zooming, channel shifting, shearing, and filling mode. As the data augmentation parameters were closely tied to the dataset, the optimal configuration for each new dataset (cropped body and cropped head) was tested and evaluated. An example of the data augmentation applied to our dataset is shown in Figure 3.

Furthermore, we compared the impact of data and resolution augmentation on the starting model. The results are presented in Table 2. In this study, the term starting model refers to a custom-designed architecture created from scratch, without the use of pre-trained models or transfer learning. It serves as an initial framework for comparison, allowing for the iterative development and evaluation of more sophisticated models. Finally, we divided our dataset into training, validation, and testing sets, and evaluated the model using accuracy, precision, and recall on the testing set. To obtain robust and reliable performance metrics, we employed 10-fold cross-validation. Our results indicated a slight performance improvement with the use of data augmentation, with accuracy increasing by 3%. Resolution augmentation led to a smaller increase in accuracy (1%). Combined, these preprocessing methods resulted in a final accuracy increase of around 4% for the starting model on the horse dataset. Given the modest performance improvement from resolution augmentation, we applied it only to the uncropped images and cropped body datasets.

**Table 2:** Starting model results on resolution and data augmentation with 10-fold validation.

Preprocessing / Metrics	Accuracy (%)	Precision (%)	Recall (%)
Starting model	60.84 ± 4.54	60.34 ± 8.74	60.34 ± 6.54
Starting model + data augmentation	63.12 ± 6.54	**64.34** ± 7.54	59.34 ± 5.56
Starting model + resolution augmentation	61.40 ± 7.25	59.12 ± 8.49	59.59 ± 6.10
Starting model + data and resolution augmentation	**63.92** ± 5.54	62.03 ± 6.42	**62.64** ± 6.98

### Performance of different models

We present the results of a Starting CNN model built using three different datasets: 1) cropped head dataset, 2) cropped body dataset, and 3) uncropped dataset. The goal was to identify which dataset yielded the best performance for the starting model.

From Table 3, we observe that the starting CNN model achieved the highest accuracy and precision on the cropped head dataset, followed by the cropped body and the uncropped dataset. The superior performance on the cropped head dataset can be attributed to the model’s ability to focus on key features related to facial recognition or head pose estimation. Similarly, the cropped body dataset provided additional features, such as the tail, that were absent in the cropped head dataset, contributing to its improved performance compared to the uncropped dataset. The uncropped dataset, which contained a significant amount of irrelevant information, likely hindered the model’s learning process

**Table 3:** Starting model results on horse, cropped body and cropped head dataset with 10-fold validation.

Dataset / Metrics	Accuracy (%)	Precision (%)	Recall (%)
Uncropped	63.59 ± 4.57	62.44 ± 8.54	58.70 ± 5.95
Cropped body	66.65 ± 8.26	63.85 ± 8.77	**62.19** ± 7.40
Cropped head	**70.48** ± 4.26	**74.35** ± 4.67	61.84 ±4.75

We further enhanced the accuracy of the model by applying transfer learning and fine-tuning. We employed several pre-trained models, including ResNet50, ResNet152, VGG16, VGG19, InceptionV3, Xception, and EfficientNetV2L, as backbone architectures. The best-performing models were achieved using VGG16 and Xception as pre-trained models. Next, we tested these two best-performing models to compare the impact of image resolution across seven different resolution sizes, as shown in Table 4. These models were built using the horse dataset without any cropping operations. The results indicated that the accuracy of both models no longer improved for resolutions below $128 \times 128$ or above $320 \times 320$. The peak performance was achieved at $128 \times 128$ for VGG16 and at $256 \times 256$ for Xception. Therefore, we set the resolution for both models to $256 \times 256$. It is important to note that the resolution was primarily increased for images below this threshold.

**Table 4:** Impact of image resolution in terms of accuracy on testing dataset using VGG16 and Xception.

Resolution / Models	($48 \times 48$)	($64 \times 64$)	($128 \times 128$)	($224 \times 224$)	($256 \times 256$)	($320 \times 320$)	($360 \times 360$)
VGG16	65.71	66.99	**70.83**	66.98	69.55	70.51	68.65
Xception	57.67	61.99	66.96	71.19	**76.97**	76.92	73.43

We compared the performance of the fine-tuned models on the cropped body dataset, as shown in Table 5. The evaluation parameters used for the comparison were accuracy, precision, and recall (on the test dataset). A total of eight models were evaluated, and the results revealed that the Xception model achieved the best overall performance. The Xception model achieved an accuracy of 78%, demonstrating its ability to classify the horse’s body with a high level of precision. However, its precision rate was 72%, which was lower than that of VGG16. Additionally, the Xception model attained a recall rate of 70%.

**Table 5:** Fine-tuning models on the body crop dataset (testing set).

Model / Metrics	Acc (%)	Precision (%)	Recall (%)
Starting model	66.65	63.85	62.19
VGG16	72.44	**76.81**	66.25
VGG19	71.79	76.47	65.00
ResNet50	61.54	63.89	57.50
ResNet152	62.82	64.86	60.00
InceptionV3	60.90	64.62	52.50
Xception	**78.010**	72.13	**69.84**
EfficientNetV2L	61.544	66.137	51.25

**Fig. 4:**
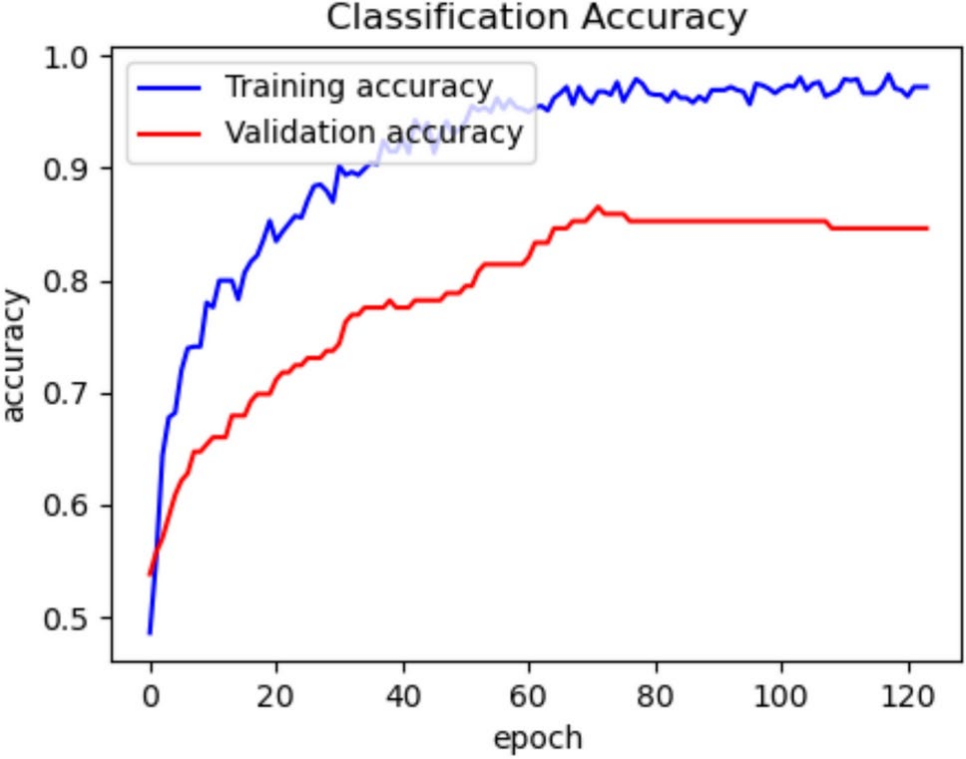
Training and validation accuracy on the cropped head dataset.

**Fig. 5:**
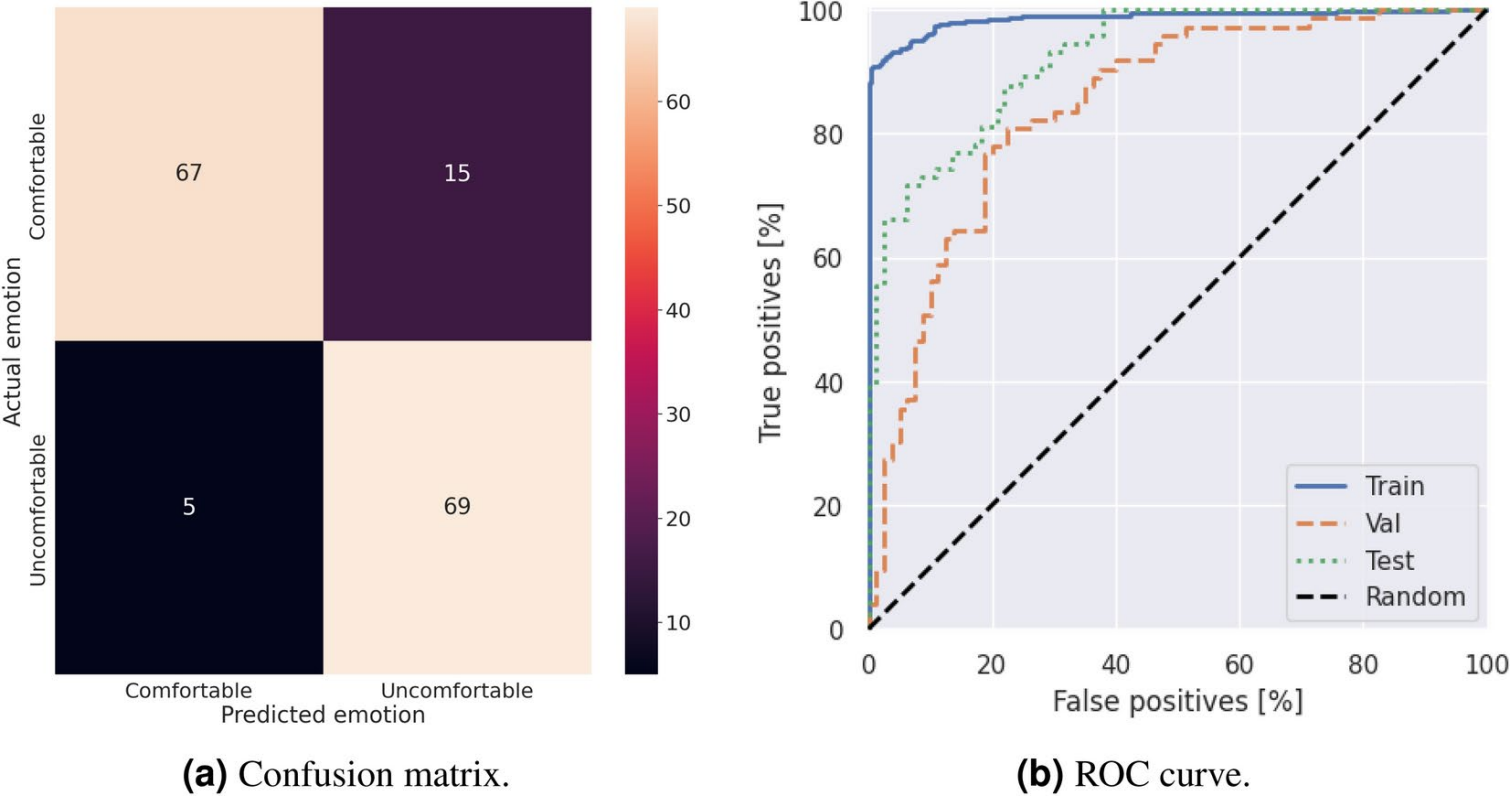
Performance on cropped head dataset.

Finally, we created a stacked model by combining the best-performing models from the previous analysis, namely VGG16 and Xception. The accuracy curves for the stacked model are shown in Figure 4. The confusion matrix is presented in Figure 5a, and the ROC curve is shown in Figure 5b. The model achieved an AUC score of 0.99 on the training set, 0.85 on the validation set, and 0.93 on the testing set.

Furthermore, we compared the performance of different models, including the starting model, fine-tuned models, and the stacked model. The results are presented in Table 6. These models were built using the cropped head and body dataset. The stacked model achieved excellent results, with an accuracy of 87%. Additionally, it achieved a precision of 79% and a recall rate of 97%, suggesting that the model was highly confident in predicting comfortable horses. These results demonstrate the superior performance of the stacked model, which outperformed the individual models.

**Table 6:** Comparison of different model on testing dataset.

Model / Metrics	Acc (%)	Precision (%)	Recall (%)
Starting model (Body)	66.65	63.85	62.19
Starting model (Head)	70.59	74.14	58.90
VGG16 (Body)	72.44	76.81	66.25
VGG16 (Head)	83.33	79.27	87.84
Xception (Body)	78.01	72.13	69.84
Xception (Head)	84.23	**84.66**	83.91
Stacking(Xc+VG) (Head)	**86.54**	79.12	**97.30**

### Interpretation of models

In this section, we discuss the regions of interest identified by the model using the LIME, SHAP, and Grad-CAM approaches, and compare these with the key features experts use to interpret emotional states. Tables 7 and 8 present comparisons of explanations based on accurate predictions of comfortable and uncomfortable emotional states. Features corresponding to these emotional states are highlighted in green and red, respectively. These explanations were derived from a model trained with the cropped head dataset.

In Table 7, we show the explanations for predictions where both the original and predicted labels are “comfortable”. We observed that the mouth and nostrils were the primary focus of the LIME explanation in both images. In the first image, SHAP highlighted the chamfer and ears, while in the second image, it emphasized the neck. Grad-CAM highlighted the neck in the first image, while in the second image, it focused on the chamfer and the background. The features identified by the expert are listed in the “True Features” column.

While each method revealed distinct regions of interest, these areas were generally consistent with expected features. Interestingly, for the explanation of a comfortable prediction with a true comfortable label, the only negative component was the horse’s chamfer behind the vertical. However, it’s important to note that there are different degrees of hyperflexion, and the further back the chamfer is, the more discomfort it causes, potentially leading to breathing issues^19^. In this case, the model still classified the horse as comfortable, and the image clearly showed only slight hyperflexion. It is possible that the model took this degree of hyperflexion into account when making its prediction, similar to the reasoning human experts use to assess a horse’s comfort.

In Table 8, we present the explanations for predictions where the model correctly identified the horse as being in an uncomfortable state. In the first row, LIME emphasized the mouth and nostrils, while SHAP focused on the background, and Grad-CAM highlighted the neck. The second image showed that Grad-CAM concentrated on the neck, SHAP emphasized the mouth, edge, and ears, while LIME focused on the mouth and ears. Despite the varied interpretations from the different methods, most of the important features were found in the background.

**Table 7:**
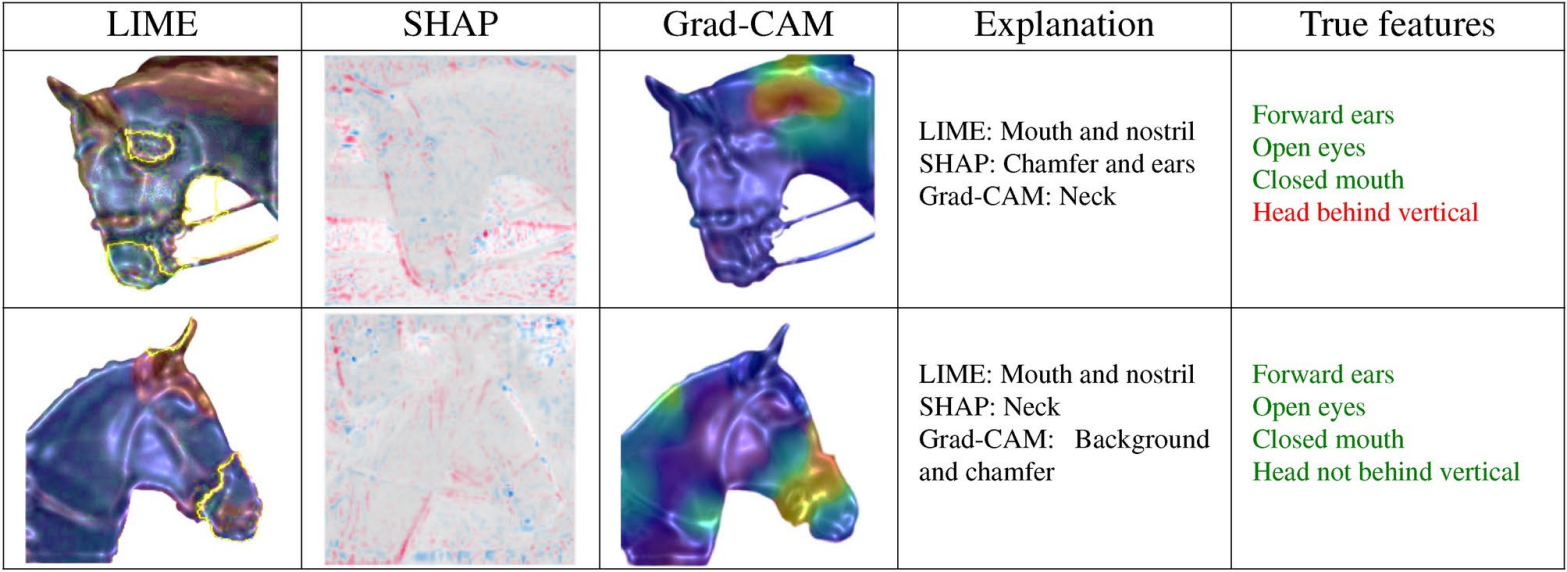
Explanation for comfortable prediction with true comfortable label.

**Table 8:**
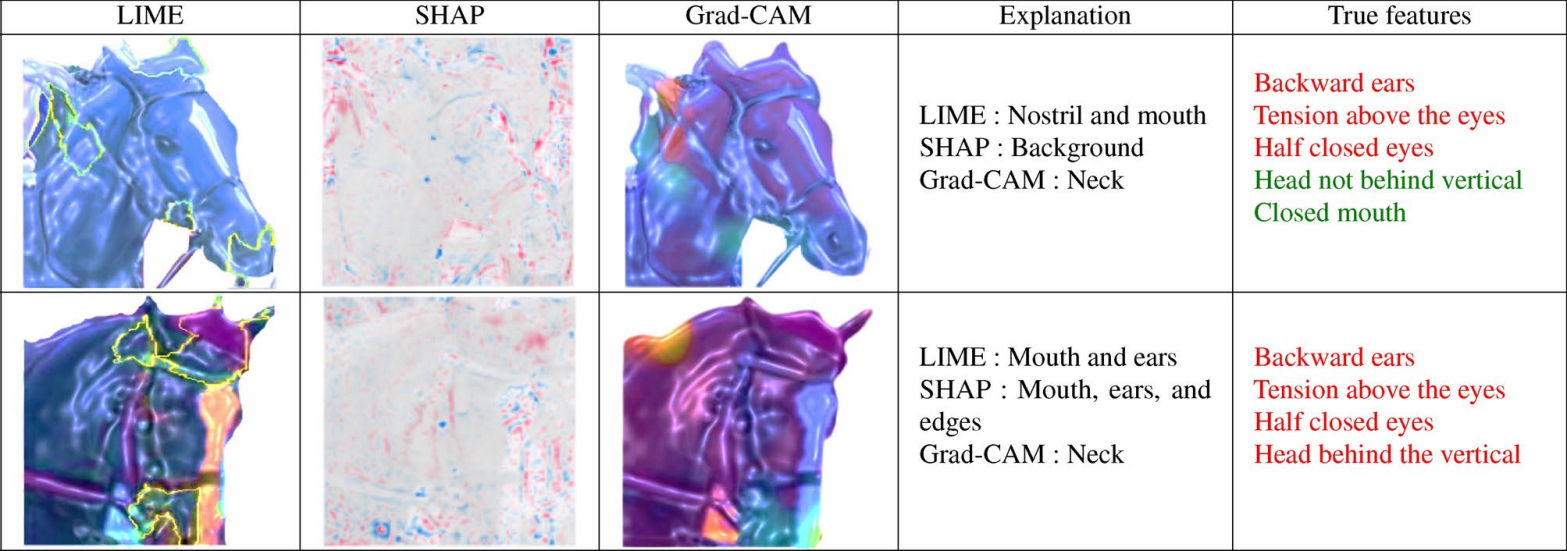
Explanation for uncomfortable prediction with true uncomfortable label.

Our findings demonstrated that the explanation approaches successfully identified key points, such as the mouth, nostrils, and chamfer, even when certain background elements were present. Interestingly, some images (shown in the first row of both Tables 7 and 8 ) contained contradictory key points, such as a closed mouth, which typically signals a comfortable condition, or a head behind the vertical, which is usually associated with an uncomfortable state. While these opposing key points appeared in both tables, it remains unclear how such features influenced the final prediction. We also observed that the key points identified by each method varied, even when applied to the same images and model. Furthermore, when compared to expert annotation methods, LIME tended to identify more accurate regions of interest than Grad-CAM and SHAP.

## Discussion

In this work, we present a novel approach based on various enhancements to the stating CNN architecture for predicting emotional states in horses. These enhancements included data augmentation, different training strategies, annotation methods, and dataset cropping, all aimed at improving the overall performance and generalizability of the model. Our annotation strategy integrated insights from two different annotation methods to construct the datasets. By incorporating preprocessing techniques such as resolution augmentation, horse body cropping, and stacking models, we were able to provide a significant performance boost to the model. Finally, we implemented several interpretation methods to explain our model’s predictions, highlighting the important features and comparing the explanations across different methods.

The model achieved an accuracy of 87% in classifying images of ridden horses as either “Comfortable” or “Uncomfortable”. Two key points are worth discussing: first, the explanations provided by the model offered insights into other areas of interest not originally included; second, the model’s underestimation of the horse’s muzzle position, which was crucial for correctly classifying ridden horses, should be addressed. Firstly, it appears that the model considered additional features beyond what was initially anticipated. Specifically, areas around the rider’s hands and reins emerged as regions of interest for predicting discomfort. This was expected, as the rider’s hand actions impact the horse’s state, particularly the condition of the mouth, a key feature in annotating horses’ emotional states. Research has shown that tension in the reins, often caused by the rider’s hand actions, leads to changes in heart rate and increased cortisol levels in horses, as well as behavioral issues like tail swishing and backward ears-features commonly associated with discomfort in the horse^17,38,39^. The model seemed to accurately classify images based on expected key points and also suggested the potential relevance of new factors, such as the rider’s influence. Secondly, one key feature the model underestimated in classifying horses as comfortable was the chamfer behind the vertical, a head-neck position known for its detrimental physiological effects. While the degree of hyperflexion in the head and neck was not considered when selecting images, research shows that such hyperflexion correlates with higher cortisol levels and discomfort^40–43^. Despite a slight chamfer behind the vertical in some images, the model classified them as comfortable, possibly due to the nuanced interpretation of head position. This highlights the potential for improving the model to detect ambiguous head-neck positions and provide more accurate predictions. Since it is difficult for the human eye to perceive these nuances, our model could therefore be enhanced to better recognize different degrees of hyperflexion^1,19^.

Despite the success of our model, there were several limitations: 1) the dataset was obtained from both private and public sources, which limits our ability to share it openly; 2) the images used were static, which might have lacked the dynamic context present in video data; 3) the dataset was small (1000 images), which might not have provided enough variation to fully train the model; and 4) the models used were not fully interpretable. To address these limitations, we have several plans for future work. First, we aim to expand our dataset by leveraging video data to generate more horse images, and manually annotate these images to enhance the dataset further. Additionally, we plan to improve the interpretability of our models. Currently, explanations are based on a small subset of images, which limits our ability to make broad generalizations about the regions of interest. In the future, we aim to identify a global set of explanations per category across different explanation methods. These explanations will be reviewed with experts to validate the key regions of interest identified by the model and potentially discover new factors that contribute to emotional state classification. Finally, we aim to develop a user-friendly interface, which, after validation, could be used by experts in their daily work. This would help avoid the manual interpretation of horse emotions, making the process more efficient and accessible.

## Conclusion

In this work, we demonstrated the use of deep learning models to predict emotional states in horses. The model trained on the cropped dataset achieved higher performance by focusing on key features. Architectural updates and improved training strategies further enhanced performance. Additionally, we showed how different interpretation methods can highlight important features, emphasizing the need for reliable model explanations due to the varying results across approaches.

## Data Availability

The image dataset used in this study is sourced from both public and private resources. Access to the public data can be provided upon request to the corresponding author.
